# Data Integration and Analysis for Medical Systems Biology

**DOI:** 10.1002/cfg.385

**Published:** 2004-03

**Authors:** Johannes H. G. M. van Beek

**Affiliations:** 1 Vrije Universiteit Faculty of Earth and Life Sciences Department of Molecular Cell Physiology De Boelelaan 1085 Amsterdam 1081 HV The Netherlands; 2 Data Integration Analysis and Logistics (DIAL) Centre for Medical Systems Biology Rotterdam and Amsterdam Leiden The Netherlands


**Comparative and Functional Genomics**
*Comp Funct Genom* 2004; **5**: 201–204.
Published online in Wiley InterScience (www.interscience.wiley.com). **DOI:** 10.1002/cfg.385

**Conference Review**

### Data integration and analysis for medical systems biology

Johannes H. G. M. van Beek*
*Data Integration, Analysis and Logistics (DIAL), Centre for Medical Systems Biology, Leiden, Rotterdam and Amsterdam, The Netherlands*

**Correspondence to:*
*Johannes H. G. M. van Beek,*
*Vrije Universiteit, Faculty of Earth and Life Sciences, Department of Molecular Cell Physiology, De Boelelaan 1085, 1081 HV Amsterdam, The Netherlands.*
*E-mail: hans.van.beek@falw.vu.nl*

Received: 7 November 2003
Revised: 21 December 2003
Accepted: 22 December 2003

**Keywords:** data integration; genomics; systems biology; databases; data mining

### Introduction

It is like listening to a stewardess in a jet airliner who is explaining the safety measures: you have heard 1000 times before that the human genome has been sequenced and that a flood of data is coming over us. The question is how the massively parallel measurements of large numbers of genes, messenger RNAs, proteins and metabolites are going to help us in prognosis and diagnosis of common human diseases. Is it a manageable problem to explain the behaviour of thousands of biomolecules from our knowledge of the molecular interactions in the cells of the human body? Can we infer from the large molecular datasets how the molecular pathways are organized and interact?

It has been argued that the life sciences are developing into a discovery- and data-driven science, with less emphasis on the hypothesis-driven experimental cycle. However, reasoning from experimentally determined facts to a well-founded theory of the underlying system is problematic. In his book on the structure of scientific revolutions, Kuhn [2] wrote, 'But though this sort of fact-collecting has been essential to the origin of many significant sciences, anyone ... will discover that it produces a morass'. Is data mining in integrated experimental databases containing large quantities of genomic and systems biology data going to produce a morass, or is this approach useful for generating hypotheses and theories which, after corroboration, lead to valid knowledge?

### Medical systems biology

Such questions are particularly important for medical systems biology. Systems biology may be defined as the study of the interactions of the large numbers of molecules (DNA, mRNAs, proteins, metabolites) that form the biological system. Systems biology combines high-density measurement methods, such as DNA chips and proteomics, with computational analysis.

The Centre for Medical Systems Biology (CMSB) in The Netherlands was opened on 1 July 2003. The CMSB is funded in the framework of a 5 year stimulation programme for genomics by the government and implemented by the Netherlands Genomics Initiative [4]. In the CMSB several medical centres (Leiden University Medical Centre, Vrije Universiteit Medical Centre, and Erasmus

Copyright © 2004 John Wiley & Sons, Ltd.

Medical Centre) collaborate with the Vrije Universiteit Amsterdam, Leiden University and the TNO Prevention and Health Research Institute, under the director Gertjan van Ommen [1].

At the CMSB, genomics and systems biology are used for identifying hidden connections between common diseases, such as Alzheimer's, depression, migraine, metabolic syndrome, vascular disease, thrombosis, arthritis, cancer and infectious diseases. Such connections between common diseases reflect underlying common biological pathways and may become manifest in the form of co-morbidity. Besides systems biology, another key approach in the CMSB is epidemiology, for which large population and patient groups, tissue sample and data collections are available.

The CMSB's systems biology research strategy is to combine measurements at several biomolecular levels (genes, gene expression, proteins and metabolites). The CMSB's working hypothesis is that interconnected changes at these vertical levels provide sensitive signatures of pathology that can be of early prognostic and diagnostic value. An even bigger challenge is to understand the measured changes in thousands of molecules simultaneously in terms of the processes inside the cell. Understanding and controlling the causal relations in the networks of intracellular signalling, transcriptional regulation and metabolism, among others, is important for understanding and influencing the progress of disease. Therapeutic interventions can then be aimed at strategically important points in the system. This goes beyond a single molecular target approach and increases the efficiency of intervention.

### DIAL (Data Integration, Analysis and Logistics)

The integration of high-density data in such a medical genomics and systems biology centre requires extensive use of computer-based approaches: integration of databases; statistical analysis of correlations amongst molecular signatures and pathology; data mining to generate hypotheses by induction; and computational analysis of pathways by relating newly measured data to external molecular and pathway databases. Therefore, the CMSB established a central project for data integration, analysis and logistics, termed DIAL.

To define interrelationships between phenotype, genotype and the intermediate biomolecular levels, linking population-based and patient-based cohort databases containing data on pathology with databases of molecular laboratory measurements (e.g. SNPs, microarrays) is a first requirement that is addressed. Further, there is a need to link the CMSB's new experimental data to external databases containing prior biological knowledge (gene annotations, pathways, etc.) to help in the interpretation of the data. Given the high data volume, the CMSB's scientists should be supported by artificial intelligence, text mining and efficient links between databases.

A fundamental question in the background is: how can valuable biological hypotheses be derived by induction from such large amounts of experimental data, avoiding Kuhn's morass? The inductive process during data mining should help to construct valuable hypotheses without creating a swamp of distracting findings reflecting noise in the data or artefacts of the data mining method.

### Knowledge by induction and data mining

At present, some life scientists seem to think that if huge masses of data are correctly stored in database systems, properly integrated and analysed, comprehensive and valid biological knowledge will emerge. This is expressed by terms such as 'discovery-driven science', as opposed to 'hypothesis-driven research'.

In the seventeenth century, Sir Francis Bacon [8] thought that if all known facts are systematically ordered, a theory of the underlying system could be derived and verified by induction. David Hume, and later Karl Popper, argued that this strategy for arriving at scientific knowledge was erroneous. True progress in science comes about by posing a hypothesis based on existing incomplete knowledge and testing the hypothesis by trying to falsify it in carefully designed experiments which yield new data to fill in gaps [3,6]. If the hypothesis could not be falsified, then the hypothesis was considered corroborated. Definitive logical proof of the correctness of a hypothesis was unattainable. However, the continuing cycle of testing of progressively refined hypotheses reflects the true nature of scientific progress, in Popper's view.

Copyright © 2004 John Wiley & Sons, Ltd. 

Given the existence of database technology for establishing and coupling large databases, many scientists now seem to expect a lot from detecting meaningful relations in the databases by computer methods. Clustering of groups of genes with similar gene expression patterns across multiple experiments is an example. However, such correlations should lead to new hypotheses and theories on the organization of the underlying biological system, which still require corroboration. Although the data-driven part of the research is very useful, the hypothesis-driven part must follow to lead to valid knowledge.

The term 'data mining' suggests that lots of rubble and rock without value will be dug up along with precious metal. Figuratively speaking, ways of separating shining nuggets of gold from the stone in which they are buried are then a prerequisite for a profitable process. If thousands of molecular changes occur, many correlations are expected based on random fluctuations. Data mining thus supports the inductive part of the scientific process: correlations are found, but it has yet to be determined whether relations are causal.

This fundamental difficulty is at present compounded by the practical problem that the higher density of data often seems to come with lower precision and accuracy. It required great care to obtain 'old-fashioned' low-density laboratory measurements, such as biochemical assays. If we perform hypothesis-driven research, a lot of attention is directed to those measurements that are critical for testing the hypothesis. If such a focus on a limited dataset is lacking, special attention is required for data reliability during mass production of high-throughput data. As is true for the mass production of goods, quality control becomes a necessary step.

In the worst case, analysis of large, heterogeneous, and to some extent unreliable, datasets might produce a much too large proportion of spurious correlations to be helpful. In the ideal case, with accurate high-throughput measurements recorded error-free in databases, the experimental work goes forward at tremendous speed, but the question is raised whether data interpretation and understanding can keep up with this. Hypotheses are easy to generate, and proliferate even faster than the data needed to critically examine them, as Robert Pirsig eloquently explained in his novel [5]. Indeed, at a recent genomics meeting, Holstege identified the challenge that in genomics the rate of generation of hypotheses is faster than the rate of verification [9]. Thus, the trouble with data analysis of high-throughput data might become that many more hypotheses can be derived from patterns in the data than can be critically examined.

### Bottom-up and top-down data mining

Analysis of large integrated databases of experimental data is going to be an inevitable development. To think of an analogy: while explorations of the earth were done in past centuries by ship, perhaps based on hypotheses of some kind ('if we go west, we will find a new route to India'), it is definitely not necessary to pose a hypothesis before starting to chart the earth with sensors and imaging equipment using satellites in orbit. However, it is not yet entirely clear how we can circumvent the limitations of the inductive mode of data mining in biomedical databases and follow this up with the necessary critical testing of the hypotheses that are generated. It becomes necessary to analyse the integrated databases, not only with inductive methods but also to test hypotheses at a high rate. The integration of inductive and deductive reasoning for data mining has been described in the context of financial and commercial data [7]. The inductive pattern discovery part is termed 'bottom-up data mining', the hypothesis-testing part was termed 'top-down data mining'.

A relevant philosophical question is whether, if the high-density molecular measurements cover a critical fraction of all the molecules in the system under study, the inductive method can to some extent replace the cycle of hypothesis falsification and formulation of improved hypotheses. Given the large number of molecules present in biological systems, it will be very difficult to keep track of the hypotheses necessary to cover so many molecular measurements. If we were to include all the molecular details, the comprehensive hypothesis would most likely be wrong in at least some of the details.

When searching on the World Wide Web it is not difficult to find statements such as 'Biology is data-driven science'. However, if the next step of critically investigating hypotheses is neglected, biology may become Kuhn's morass. Thus, the development of top-down data mining, i.e. hypothesis testing, for the analysis of high density biological data is important.

Copyright © 2004 John Wiley & Sons, Ltd. 

### Not the trees, but the forest

With regard to the multiple hypothesis testing problem, where a large number of false positive answers arise when a huge number of tests is performed simultaneously, there may be various answers. Some degree of coarse graining may be helpful for some questions. The measurement of the level of a single molecule often does not yield the answer, because it is an interconnected parallel change in many molecules belonging to a pathway. When correlated changes between two molecules appear while testing many possible combinations of molecules, this may be due to random fluctuations, but when many molecules belonging to the same pathway change in a certain direction this provides a more reliable signature of a meaningful change in the system. Therefore, at the CMSB such interconnected changes will be used for prognosis, diagnosis and classification of disease.

Alternatively, one can concentrate on large correlations or changes whose magnitude is such that on statistical grounds less than one instance of at least that likelihood is found in the total integrated dataset under the null hypothesis, i.e. without a real underlying change or relation. This is analogous to using small E-values for selecting sequence alignments from a BLAST search. For large datasets this is a much more stringent criterion than the common criteria for significance (traditionally $p < 0.05$ or $<0.01$). However, the E-value has great practical value: if there is one true relation in the dataset and the 'E-value' used is 10, the ratio of true to false positives is 1 to 10. The task of weeding out the false positives becomes uncomfortably large at high 'E-values'.

To analyse the data, it is particularly worthwhile to investigate how much of the measured changes can be predicted from reliable prior biological knowledge, preferably formalized in a computational model. If debatable assumptions have to be introduced into the model to explain measured data, these constitute new hypotheses to be tested with new results. Critical re-examination of measured data is sometimes also indicated and helps in data quality control. It will be a big challenge for the future to build a reliable model for sizable parts of the whole biomolecular system.

### Conclusion

Integration of databases containing experimental data in genomics and systems biology is going to be an inevitable development. When accurate high-throughput measurements speed up the experimental part of the scientific discovery cycle, the interpretation and analysis part of the scientific process will become more limiting. Many data-mining techniques for use on the integrated databases are inductive in nature and may help the formulation of hypotheses. However, creative scientific reasoning, the design of new experiments, and critical testing of hypotheses, theories and computational models remain of vital importance now that data collection is increased in scale.

### Acknowledgements

Work in the Centre for Medical Systems Biology is supported by a Centre of Excellence grant from the Netherlands Genomics Initiative.

### References

1. Centre for Medical Systems Biology: **http://www.cmsb.nl**
2. Kuhn TS. 1970. *The Structure of Scientific Revolutions*, 2nd edn. University of Chicago Press: Chicago, IL.
3. Magee B. 1973. *Popper*. Collins: London.
4. Netherlands Genomics Initiative: **http://www.genomics.nl**
5. Pirsig RM. 1974. *Zen and the Art of Motorcycle Maintenance*. Corgi Books: London.
6. Popper KR. 1976. *Unended Quest*. Library of Living Philosophers. Fontana: London.
7. Simoudis E, Livezey B, Kerber R. 1996. Integrating inductive and deductive reasoning for data mining. In *Advances in Knowledge Discovery and Data Mining*, Fayyad UM, Piatetsky-Shapiro G, Smyth P, Uthurusamy R (eds). AAAI Press: Menlo Park, CA; and MIT Press: Cambridge, MA; 353–373.
8. Sir Francis Bacon. 1620. *The New Organon or True Directions Concerning the Interpretation of Nature*: **http://www.constitution.org/bacon/nov_org.htm**
9. Wixon J, Marsh J. 2003. Meeting Report: ESF Programme on Functional Genomics 1st European Conference: Functional Genomics and Disease. *Comp Funct Genom* **4**: 549–557.

Copyright © 2004 John Wiley & Sons, Ltd. 
